# Prokaryotic defense systems: Diversity and evolutionary adaptation

**DOI:** 10.1002/mlf2.70068

**Published:** 2026-02-21

**Authors:** Changjialian Yang, Luyao Gong, Jing Guo, Hua Xiang

**Affiliations:** ^1^ State Key Laboratory of Microbial Diversity and Innovative Utilization, Institute of Microbiology Chinese Academy of Sciences Beijing China; ^2^ University of Chinese Academy of Sciences Beijing China; ^3^ Tianjin Institute of Industrial Biotechnology Chinese Academy of Sciences Tianjin China

**Keywords:** ancestral immunity, defense systems, diversity, evolutionary adaptation, prokaryotes

## Abstract

Bacteriophages and archaeal viruses are the most abundant biological entities on Earth. Through a long‐standing co‐evolutionary arms race, they have driven the emergence of a diverse repertoire of prokaryotic defense systems. This review summarizes these systems, highlighting their diverse antiviral mechanisms across distinct stages of viral infection, from surface barriers and inducible innate responses to specific adaptive defenses, and the intricate interplay between these defense strategies. By examining host–virus counter defense dynamics, the trade‐off between survival benefit and adaptive cost, the co‐evolution of RNA and protein components, and the comparison with eukaryotic immune systems, we underscore the intrinsic complexity and evolutionary plasticity of prokaryotic antiviral immunity. A deeper understanding of these processes and mechanisms will not only shed light on the origins and evolution of the immune system but also provide valuable opportunities for the development of biotechnological tools.

## INTRODUCTION

Bacteriophages and archaeal viruses are among the most abundant biological entities on Earth and show strict host specificity for prokaryotes. The global bacterial population is estimated to be approximately 10^30^, whereas phage particles are thought to outnumber them by nearly an order of magnitude^1^. Phages greatly enhance horizontal gene transfer (HGT) and drive a long‐standing co‐evolutionary dynamic that has shaped the genomic diversity of bacteria over billions of years.

Prokaryotes have evolved a diverse array of defense systems that disrupt infections at multiple stages^2^. For instance, prokaryotes may block phage recognition or genome injection through surface barriers^3^, ^4^, interrupt infection by degrading or blocking the replication of phage genomes through various innate immune systems^5^, ^6^, use adaptive immune systems to memorize and rapidly eliminate previously encountered phages^7^, or, if all defense systems fail, trigger abortive infection (Abi) to sacrifice the infected host cell to prevent the further spread of viruses^8^. The evolutionary pressure exerted by phages drives the continuous emergence and maintenance of a broad range of prokaryotic‐defense strategies^9^.

Recent advances in high‐throughput methodologies, computational algorithms, and artificial intelligence have improved the ability to identify and characterize defense systems on a genome‐wide scale. Analysis of bacterial and archaeal defense systems revealed that these systems are frequently associated with mobile genetic elements (MGEs) and usually clustered within specific genomic regions. This has led to the concept of “defense islands,” in which protein families located near known defense genes are likely to belong to novel defense systems or contribute to immune function^10^. Synergistic interactions between defense systems may promote their colocalization within genomes and facilitate HGT across species. Once a few defense systems are known, many more can be identified and characterized across prokaryotes based on sequence homology, structural similarity, and genomic regions^11^, ^12^, ^13^, ^14^. Besides the well‐known clustered regularly interspaced short palindromic repeats (CRISPR)–CRISPR‐associated (Cas) system^15^, ^16^, recent studies have revealed numerous novel defense systems involving enzymes such as NADases, helicases, ATPases, and deaminases, highlighting the critical role of protein complexes in prokaryotic defense. Notable examples include Septu^17^, Gabija^18^, Thoeris^19^, ^20^, restriction by an adenosine deaminase acting on RNA (RADAR)^12^, ^21^, DUF4297‐Her^22^, Nezha^23^, Shedu^5^, and Hachiman^24^ (Table 1). The rapid expansion of mechanistic studies on these antiphage systems has provided valuable insights into how microbes counter environmental and viral challenges.

**Table 1:** Structural and functional characteristics of novel prokaryotic defense systems.

System	Mechanism	Components	Domain	Structure	Molecular mass	References
Septu	Degradation of phage genome	PtuA, PtuB	ATPase, nuclease	PtuA‐PtuB complex(6:2)	~380 kDa	17
Gabija	Abi induces cell death	GajA, GajB	ATPase, helicase	GajA‐GajB complex(4:4)	~500 kDa	18
Thoeris	Abi induces cell death	ThsA, ThsB	NADase, TIR	ThsA tetramer	/	19,20
DUF4297‐Her	Degradation of phage genome	DUF4297, HerA	ATPase, helicase	HerA‐DUF4297 complex(6:12)	~1 MDa	22
Nezha	Degradation of phage genome	Sir2, HerA	NADase, helicase	HerA‐Sir2 complex(6:12)	~1 MDa	23
Shedu	Degradation of phage genome	SduA	Nuclease	SduA tetramer	/	5
RADAR	Inhibition of phage propagation	RdrA, RdrB	ATPase, adenosine deaminase	RdrA‐RdrB complex(7:12 or 14:12)	~10 MDa	12, 21
Hachiman	Degradation of host and phage genome	HamA, HamB	Nuclease, helicase	HamA‐HamB complex(1:1)	/	24

Abi, Abortion infection; RADAR, restriction by an adenosine deaminase acting on RNA; TIR, Toll/interleukin‐1 receptor.

This review analyzes the diverse mechanisms of prokaryotic defense systems that operate at distinct stages of viral infection and the complex interactions among them. It also highlights the multifaceted evolutionary adaptability of the prokaryotic immune system, emphasizing the intrinsic complexity and dynamic interplay between these systems. By elucidating modular conservation and directional evolution, we uncover previously unrecognized connections and propose a refined framework for understanding the diversity and adaptive capacity of prokaryotic defense systems.

## DIVERSITY OF DEFENSE SYSTEMS

Prokaryotes and their viruses (bacteriophages and archaeal viruses) engage in continuous evolutionary competition driven by environmental changes and infection pressure^2^. Over evolutionary time scales, prokaryotes have evolved defense systems that target distinct stages of the viral life cycle, enabling them to adapt to environmental changes and resist foreign invasion.

**Figure 1:**
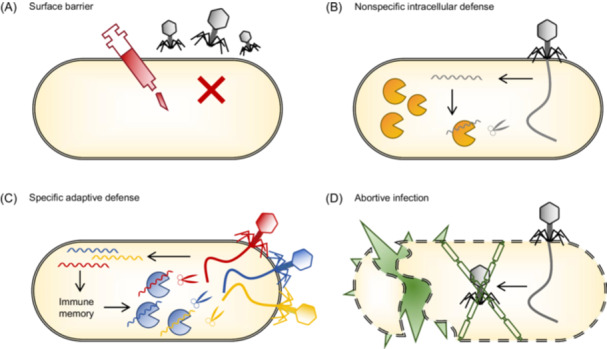
Diversity of prokaryotic defense mechanisms. (A) Surface barrier. Its main role is to prevent viral attachment to the host and block the injection of the viral genome. By preventing the virus from entering the cell, this line of defense acts as an initial shield against infection. (B) Nonspecific intracellular defense. This system functions by degrading the genetic material of the invading viruses or preventing their replication. It provides broad, nonspecific protection by targeting a wide range of pathogens without requiring prior exposure. (C) Specific adaptive defense. This system specifically recognizes foreign genetic material based on previous encounters and efficiently targets and eliminates viruses. (D) Abortion infection (Abi). When all other defense systems fail, Abi can act at that point to prevent the spread of infection by inducing cell death.

Defense systems function by interfering with the interactions between phages and prokaryotes, destabilizing viral structural components, and inhibiting phage DNA replication. Escape mutations frequently occur in key phage genes involved in DNA replication, structural assembly, and host takeover mechanisms^25^. Prokaryotes possess an array of defense systems that recognize and eliminate invaders and enhance their resistance to future infection. These defense systems can be broadly categorized into surface barriers, nonspecific intracellular defenses, and specific adaptive defenses (Figure 1).

### Surface barriers

**Figure 2:**
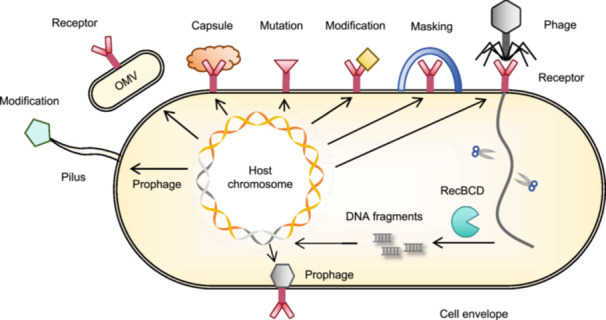
Surface barriers preventing phage attachment and entry. Bacteria block phage binding by modifying their receptors through masking, mutations, or modifications. Outer membrane vesicles (OMVs) can act as decoys to prevent viral recognition and attachment. Some phages integrate their genomes into bacterial chromosomes as prophages, encoding defense genes that modify key cell envelope components to block further phage invasion. Archaea have a proteinaceous S‐layer composed of glycosylated proteins that forms a primary barrier to impede viral attachment and penetration.

Surface barriers play a crucial role in inhibiting viral attachment and genome injection (Figure 2). The barriers to bacteria rely on cell envelope and biofilm formation^4^. The cell membrane contains specific surface receptors that interact with viral receptor‐binding proteins (RBPs). Bacteria use receptor masking, modification, and mutation to block viral attachment. For example, *Pseudomonas aeruginosa* glycosylates its pili to prevent viral binding^26^. Some phages integrate their genomes into bacterial chromosomes to form prophages encoding defense proteins that alter key membrane components to block subsequent infections. For instance, the prophage D3112 of *P. aeruginosa* produces proteins that interact with ATPase to inhibit pilus extension^27^. Proteins of the bacterial outer membrane, which maintain membrane stability and participate in diverse biological processes, also contribute to phage defense^28^. Biofilms, which comprise high‐molecular‐weight polymers and other components, form a protective layer that shields bacteria from antibiotics and other invaders. Extracellular vesicles (EVs) or outer membrane vesicles (OMVs) containing surface receptors can act as molecular decoys, diverting phage recognition from the host cell^29^.

Archaea have evolved distinct surface defense mechanisms that rely on specialized cell envelope structures^30^. Unlike bacteria, many archaea are surrounded by a proteinaceous S‐layer composed of glycosylated proteins, which serves as the primary barrier against viral entry by creating a complex surface that impedes viral attachment and penetration^30^, ^31^. The diversity of S‐layer protein composition further complicates viral invasion, making it difficult for viruses to recognize and breach the archaeal cell envelopes. In addition, archaeal membranes contain unique lipids that provide an extra layer of defense against viral entry^32^. Filamentous glycans extending from the S‐layer form an additional glycan barrier, collectively establishing a multilayered defense architecture that effectively reduces the likelihood of successful infection^30^.

### Nonspecific intracellular defense

**Figure 3:**
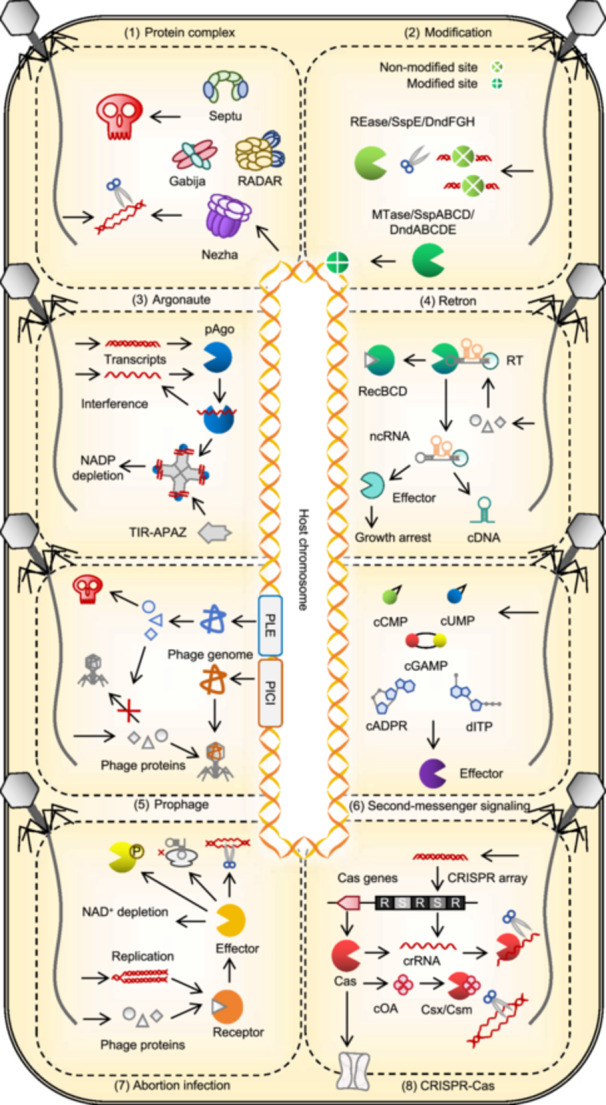
Key intracellular antiviral defense mechanisms in prokaryotes. (1) Defense‐related protein complex. Large protein complexes composed of enzymes found in defense islands contribute to diverse bacterial‐defense strategies. (2) Restriction–modification system. Foreign DNA is cleaved, whereas modified host DNA is protected through sequence‐specific modification. (3) Argonaute system. Some prokaryotic Argonaute (Ago) proteins cleave small molecules, such as NAD^+^/NADP^+^, whereas others are associated with nucleases that degrade nucleic acids in a guide‐dependent or nonspecific manner. (4) Retron system. This system produces cDNA and triggers growth arrest after phage attack. (5) Prophage‐mediated defense. Prophages are genomic elements that use phage components to limit or block their replication. (6) Second‐messenger signaling system. Phage infection induces the production of signaling molecules that trigger effectors, leading to growth arrest, cell death, or inhibition of phage replication. (7) Abortive infection (Abi) system. Infected cells undergo self‐destruction to prevent phage propagation, often in coordination with other defense systems. (8) CRISPR–Cas system. Phage DNA fragments are captured as spacers and later used to guide Cas proteins to destroy future invaders. APAZ, analog of Piwi‐Argonaute‐Zwille; PICI, phage‐inducible chromosomal island; PLE, phage inducible chromosomal islands‐like element; TIR, Toll/interleukin‐1 receptor.

After bypassing the surface defenses, viruses inject their genetic material into host cells (Figure 3). Viral genomes, which are typically linear and lack protective DNA‐binding proteins, are particularly vulnerable to broad‐spectrum, intracellular defense systems.

#### Small‐molecule arsenal

Bacteria can defend themselves against phages using small molecules that interfere with the phage life cycle. For instance, *Streptomyces* species produce secondary metabolites that block phage replication by intercalating into phage DNA^33^, ^34^, ^35^. One such example is daunorubicin, a DNA‐intercalating agent^34^. Upon infection, the phage injects its DNA into the host, which then replicates for the propagation of the virus. Daunorubicin intercalates between DNA base pairs, distorting the phage genome and preventing circularization or interaction with essential replication proteins^35^. Consequently, the replication of phage DNA is halted. Another compound, doxorubicin, functions similarly and inhibits the replication of various double‐stranded DNA (dsDNA) phages^34^. These molecules exert minimal toxicity toward host bacteria, allowing bacterial growth to continue, whereas the phage is neutralized, providing a selective advantage for bacteria in diverse environments^35^.

These small molecules are distinct from protein‐based systems because of their broad‐spectrum antiviral activity^34^. Targeting a fundamental step in the phage life cycle, DNA replication protects bacteria against a wide range of dsDNA phages. Moreover, these metabolites can diffuse within microbial communities, offering collective or population‐level protection^10^, ^34^. Given the widespread production of such compounds by *Streptomyces* and other bacteria, chemical defense mechanisms likely play a crucial evolutionary role in shaping microbial community structures through robust and versatile antiphage activity.

#### Host modification systems

Restriction–modification (R–M) systems, found in approximately 83% of prokaryotes^36^, comprise a restriction endonuclease (REase) that recognizes and cleaves specific DNA sequences and a methyltransferase (MTase) that protects host DNA through methylation. The host DNA is methylated after replication to avoid cleavage, whereas unmethylated foreign DNA is degraded by REases. R–M systems are classified into four types, with type IV systems lacking MTase activity and targeting modified DNA^37^. Numerous viruses use 7‐deazaguanine modification in their genomes to avoid R–M recognition and cleavage^38^.

Bacteriophage exclusion (BREX) system, which is closely related to R–M systems, is found in 10% of microbial genomes and is divided into six subtypes^39^. BREX methylates host DNA but does not cleave foreign DNA, likely restricting phages through alternative mechanisms such as blocking viral replication or interfering with essential viral proteins^39^. Unlike traditional R–M systems, BREX uses non‐palindromic methylation patterns, suggesting a more complex mode of defense involving potential protein–protein interactions.

Defense island system associated with restriction–modification (DISARM) is another related system. It comprises five genes, methylates its own cytosine residues, and provides a broad‐spectrum viral defense^39^, ^40^. Unlike canonical R–M systems, DISARM uses a multiprotein complex for DNA restriction^40^.

Phosphorothioation (PT)‐based R–M systems are a recently recognized class of R–M systems in which self‐modification is not methylation but a sulfur atom substituted for a non‐bridging oxygen in the phosphodiester backbone^41^. In the Dnd system, the DndABCDE cluster mediates PT modification of self‐DNA, whereas the DndFGH cluster restricts unmodified foreign DNA^42^. These modules function independently of one another. The DndFGH module alone encodes restriction, DNA binding, and ATP‐dependent helicase domains, establishing it as a standalone prokaryotic immune system^43^. Ssp system, which comprises the modification module SspABCD and the restriction module SspE, works with the Dnd system to provide broad‐spectrum viral resistance^42^. The Dnd system modifies dsDNA, whereas the Ssp system acts on single‐stranded DNA (ssDNA). Methylation of viral DNA does not hinder DndFGH‐mediated restriction, suggesting possible cooperation between classical R–M and PT systems in viral defense.

#### Argonaute systems

Prokaryotic Argonaute (pAgo) proteins, found in approximately 10% of bacteria and one‐third of archaea^36^, play a critical role in inhibiting the invasion of plasmids and viruses^44^. These Argonaute (Ago) proteins are homologous to their eukaryotic counterparts, but lack the accessory factors required for RNA interference (RNAi)^45^. pAgo systems acquire their guides from degraded fragments of invading DNA or RNA, which are usually generated by host nucleases such as RecBCD or AddAB^46^, ^47^, ^48^. Unlike CRISPR–Cas, pAgo lacks immune memory and relies on transient fragments for defense.

pAgos are categorized into long and short groups, each with distinct domain architectures and mechanisms^46^, ^49^. Long pAgos possess a four‐domain structure (N‐PAZ‐MID‐PIWI). Unlike CRISPR–Cas, they cannot unwind and cleave dsDNA and use either ssDNA or RNA guides to target and cleave complementary foreign ssDNA or ssRNA, restricting phage or plasmid replication^46^, ^49^. In contrast, short pAgos have a streamlined architecture (MID‐PIWI) and lack the catalytic activity required for cleavage^46^. However, short pAgos often possess APAZ domains, similar to the N domain of long pAgos, and are typically fused with other functional domains, such as SIR2 or Toll/interleukin‐1 receptor (TIR)‐like domains^46^. A representative example is the SPARTA system, in which a short pAgo forms a heterodimer with the TIR and APAZ domains^6^, ^50^, ^51^. This complex binds to the target ssDNA using small RNA guides, and oligomerizes to activate the NADase activity of the TIR domain, reducing NAD^+^ levels and inducing Abi activity^50^.

#### Reverse transcriptase (RT)

Prokaryotic retrons represent a branch of RT‐based defense systems composed of an RT, a noncoding RNA (ncRNA) that gives rise to multicopy ssDNA (msDNA), and a cognate effector protein^12^, ^52^. Rather than executing defenses through a universal growth arrest mechanism, retrons form tripartite complexes in which msDNA directly interacts with the effector, and phage‐triggered perturbations activate effector functions to inhibit viral replication. For instance, in the well‐studied Ec86 retron, RT and msDNA assemble with a ribosyltransferase effector to form a supramolecular filament^53^, ^54^. Phage‐encoded Dcm methylation of msDNA activates this filament, unleashing effector‐mediated NAD(P)^+^ hydrolysis and inhibiting infection^53^. By contrast, the Ec83 Retron–Septu system uses an “arrest‐and‐release” strategy: under normal conditions, msDNA sequesters the PtuAB nuclease complex in an autoinhibited state, whereas phage infection promotes msDNA degradation, releasing PtuAB to degrade ssDNA and restrict phage propagation^55^. Thus, retron defense relies on effector‐specific activation mechanisms, with msDNA acting as a molecular switch.

Defense‐associated reverse transcriptase (DRT) constitutes a distinct class of enzymes that offer robust protection against dsDNA phages. DRT systems typically synthesize cDNA from an ncRNA template during infection, producing dsDNA with an open reading frame (ORF)^56^, ^57^. Translation of this ORF triggers growth arrest and halts the viral replication cycle. DRT systems are categorized into three classes based on their structure and function^58^. These systems illustrate how MGEs can defend against phages and drive genetic innovation^57^.

#### Prophage‐induced chromosomal islands (PICIs)

Phages can integrate their genes into host genomes as prophages, which play roles in HGT and phenotypic modulation (e.g., motility, antibiotic resistance, and metabolism) and serve as hotspots for defense systems^59^, ^60^. PICIs limit phage proliferation by exploiting phage proteins for self‐packaging during infection^61^. PICI genomes (10–15 kb) assemble more efficiently than invading phage genomes but cannot completely inhibit replication. PICIs encode immune systems, such as MazF‐like (SMA) and higher eukaryotes and prokaryotes nucleotide binding transmembrane domain (HEPN‐TM), which provide broad defense. They also target MGEs by blocking HGT^62^. Phage‐induced chromosomal island‐like elements (PLEs), which are closely related to PICIs, are found in *Vibrio cholerae* and block phage replication and assembly by utilizing phage proteins.

#### Second‐messenger signaling systems

Cyclic oligonucleotide‐based antiphage signaling (CBASS) systems represent a major class of nucleotide‐based defense systems that can directly sense phage invasion and subsequently activate multiple effectors. CBASS is found in over 10% of prokaryotic genomes, with diverse effectors contributing to phage resistance^63^. CBASS sensors detect phage DNA or other molecular patterns and produce cyclic GMP–AMP (cGAMP) signaling molecules that activate various effectors^63^. These effectors include phospholipases, transmembrane proteins, nucleic acid‐degrading domains (HNH in Cap5 and REase in Cap4), and NAD^+^‐degrading domains (TIR‐SAVED). This self‐destructive cascade prevents the replication of phages.

Pyrimidine cyclase system for antiphage resistance (Pycsar) system uses pyrimidine cyclase PycC to produce 3′,5′‐cyclic cytidine monophosphate (cCMP) and 3′,5′‐cyclic uridine monophosphate (cUMP) as second messengers^64^. Upon phage detection, Pycsar enzymes synthesize cCMP and cUMP, which activate effectors that trigger cell death and prevent phage replication. Pycsar specifically uses cyclic pyrimidines to minimize interference with other cellular processes regulated by cyclic nucleotides such as cAMP.

Thoeris system also relies on nucleotide‐derived signaling but uses a distinct signaling molecule. Type I Thoeris systems, comprising ThsB and ThsA proteins, use ThsB to sense infection and generate 1″–3′ glycocyclic ADP‐ribose (gcADPR), which binds to the SLOG domain of ThsA and promotes filament assembly^19^. This structural change activates the SIR2 domain of ThsA, leading to rapid NAD^+^ depletion and halting of viral replication^19^, ^65^. Filament formation ensures efficient phage resistance^66^. Type II Thoeris system expands this mechanism through an unconventional signaling molecule, histidine‐ADP‐ribose (His‐ADPR), generated by TIR domain‐containing proteins. His‐ADPR binds to the C‐terminal macrodomain of ThsA, inducing ThsA oligomerization and disrupting the bacterial cell membrane, ultimately triggering cell death and blocking phage infection^67^.

Kongming system uses a non‐canonical nucleotide, deoxyinosine triphosphate (dITP), as a second messenger, expanding the diversity of nucleotide‐based immunity^68^. KomA, phage deoxynucleotide monophosphate kinases (DNKs), and host nucleoside diphosphate kinases (NDKs) collaborate to synthesize dITP from phage‐derived dAMP, avoiding self‐toxicity through substrate specificity and metabolic regulation^68^. dITP binds to KomB, activating KomC to deplete NAD^+^ and trigger immunity^68^. Phages counteract this using Dmp enzymes (for dAMP depletion) and delaying DNK expression to block dITP synthesis^68^. This system introduces noncanonical nucleotides as immune signals, expands nucleotide‐based defense paradigms, and links them to eukaryotic mechanisms of action.

#### Toxin–antitoxin (TA) systems and Abi

TA systems exemplify the principle of prokaryotic antiviral defense, which couples molecular sensing with self‐elimination. They are broadly involved in stress responses, persistence, and biofilm formation and contribute to antiviral defense^69^. During phage infection, antitoxins are often destabilized or degraded, releasing toxins that cleave RNA or DNA, inhibit translation, or disrupt essential metabolic pathways, leading to growth arrest and/or cell death. Nucleases, particularly ribonucleases, are among the most prevalent toxins that cause rapid and efficient lethality^70^. TA modules are frequently embedded within defense islands and share extensive domain shuffling and functional overlap with other Abi‐associated systems^71^.

The distinction between Abi and TA is not clear‐cut. Abi typically involves distinct sensor–effector modules, in which sensors detect phage components and transmit activation signals to effectors that trigger abortive responses^72^. These responses include the inhibition of essential cellular processes (e.g., translation, transcription, and replication), degradation of key metabolites such as NAD^+^, and induction of membrane depolarization, ultimately leading to cell death and prevention of viral propagation^64^, ^73^, ^74^, ^75^. For example, the RADAR system converts ATP into ITP, accumulating inosine mononucleotides that disrupt viral replication by disturbing nucleotide homeostasis and inducing metabolic imbalance^12^, ^21^. The Zorya system uses proton channels to depolarize membranes and induces cell death or metabolic arrest to prevent phage replication^65^. Thus, recognizing Abi as a population‐level defense strategy rather than a standalone mechanistic class indicates that different systems use diverse sensors and execution modules while converging on a common defensive outcome.

### Specific adaptive defense: CRISPR–Cas systems

Specific adaptive defense systems possess memory and adaptability, allowing rapid and specific responses based on the type and abundance of pathogens (Figure 3). Among prokaryotic immune mechanisms, only CRISPR–Cas systems show programmable adaptation, storing fragments of invading genetic material as molecular memories in CRISPR arrays.

#### Mechanism of CRISPR–Cas systems

CRISPR–Cas systems are found in approximately 50% of bacteria and 87% of archaea^16^. They are classified into two classes, over six types, and more than 30 subtypes^76^. The defense process involves three stages: adaptation, expression, and interference. During adaptation stage, fragments of invading DNA, called protospacers, are integrated into CRISPR arrays, forming heritable immune memory. Upon reinfection, CRISPR arrays are transcribed and processed into mature CRISPR RNAs (crRNAs), which guide Cas proteins to recognize and cleave target nucleic acids^77^. In Type II CRIPSR–Cas systems, trans‐activating CRISPR RNA (tracrRNA) assists in processing long precursor transcripts into mature crRNAs^78^.

During the adaptation stage, CRISPR memory is established via two pathways: naïve and primed. Both require the Cas1–Cas2 complex; however, primed adaptation also depends on a pre‐existing spacer that partially matches the invasive DNA, enhancing the acquisition efficiency^79^, ^80^. In naïve adaptation, foreign DNA is processed by host double‐strand break repair enzymes (AddAB or RecBCD)^7^, ^79^ and captured as spacers by the Cas1–Cas2 complex^81^, ^82^. Primed adaptation is directed by a pre‐existing spacer to related viruses; thus, it is more efficient and avoids self‐targeting. In some organisms, such as *Haloarcula hispanica*, the naïve pathway is inactivated to prevent self‐DNA acquisition^83^, whereas primed adaptation tolerates protospacer adjacent motif (PAM) mutations up to 23 variants^84^, ensuring effective non‐self‐discrimination while preventing viral evasion.

After adaptation, the expression and interference activate the immune system. During expression stage, precursor crRNAs are processed into mature crRNAs using Cas6 in most class 1 systems, RNase III in Type II systems, and Cas12 or Cas13 in some class 2 systems^85^. During interference guided by crRNAs, Cas protein complexes, such as the Cas complex for antiviral defense (Cascade) in class 1, or single effector proteins, such as Cas9, Cas12, or Cas13 in class 2, bind to and cleave foreign nucleic acids. Type III systems produce cyclic oligoadenylate (cOA) to activate different types of effectors, such as nucleases, proteases, or adenosine deaminases, leading to cell dormancy^86^, ^87^. In addition, certain CRISPR–Cas systems exert antiviral activity via transcriptional inhibition of target genes (e.g., Cas12m)^88^.

#### CreTA safeguard systems

Although CRISPR–Cas systems are effective, they face viral countermeasures, most notably anti‐CRISPR (Acr) proteins that inhibit Cas effectors^89^. This has driven the evolution of CreTA, a TA–RNA pair associated with CRISPR loci that protects CRISPR–Cas integrity and regulates its activity^90^. The small RNA toxin CreT sequesters the rare arginine tRNA^UCU^, blocking protein synthesis and exerting a bacteriostatic effect. The RNA antitoxin CreA prevents CreT toxicity by repressing transcription via the Cascade complex. When viral Acr proteins disable the CRISPR–Cas systems, the protective function of CreTA is unleashed as a fail‐safe mechanism, triggering cell death^90^. In addition, CreR, a mini‐CRISPR regulatory RNA analogous to CreA, regulates CRISPR–Cas activity by sensing changes in crRNA abundance and modulating Cas protein expression to counteract Acr protein inhibition^91^.

Through these auxiliary modules, the CRISPR–Cas system co‐evolves with TA–RNA pairs to maintain immune and functional integrity. It can circumvent Acr inhibition by modifying the regulatory pathways, thus preserving immune function. These findings extend the current understanding of self‐regulation and antiviral resilience of CRISPR–Cas system and inspire new strategies for antibacterial applications, including targeting multidrug‐resistant pathogens (MDRs)^92^.

#### CRISPR–Cas ancestors

The evolutionary origins of CRISPR–Cas systems can be traced to two major ancestral lineages, IscB and TnpB nucleases, which are classified as Obligate Mobile Element Guided Activity (OMEGA) systems^93^. The localization and mobility of these transposon‐encoded nucleases likely influence their RNA guide specificity, suggesting multiple independent origins. Specifically, IscB proteins (originating from IS200/IS605 transposons), which are considered evolutionary precursors of Cas9, use ncRNA to guide dsDNA cleavage^93^. Similarly, TnpB proteins constitute the ancestor of Cas12^94^. By contrast, AbiF, a Type III TA system with a conserved ncRNA antitoxin, is a precursor of Cas13^95^. Unlike OMEGA systems with inherent RNA‐guided mechanisms, AbiF lacks RNA‐targeting specificity, moving to CRISPR–Cas13, a rare evolutionary leap. These findings provide distinct paths for CRISPR–Cas evolution: transposon‐derived OMEGA systems (Cas9/12) and TA‐derived systems (Cas13), highlighting how innate immune components can evolve into adaptive defenses during the host–pathogen arms race^96^.

### Synergistic defense networks

The prokaryotic immune system achieves a multilayered defense by integrating innate and adaptive components. Ago systems degrade foreign nucleic acids but lack memory; however, their fragments serve as precursors for CRISPR spacers, linking nucleic acid‐guided degradation to adaptive memory^7^. Moreover, R–M systems enhance CRISPR spacer acquisition by generating DNA breaks, enhancing immune efficiency. Type I R–M systems promote Type VI CRISPR acquisition^97^, and in *Streptococcus thermophilus*, Type II CRISPR–Cas and Type II R–M systems synergize to strengthen the defense.

By integrating orthogonal defense modules and layered redundancy, prokaryotic defense networks achieve robustness against viral evolution while minimizing single‐point vulnerabilities. Retrons assist CRISPR adaptation by generating cDNA for new spacers^52^, ^98^. The CRISPR Cascade complex inhibits the toxin CreT. When the CRISPR Cascade is inactivated, CreT sequesters the rare arginine tRNA^UCU^, halting cell growth^99^.

These individual adaptations scale up to population‐level resilience through optimized resource cooperation. Type I and Type III CRISPR–Cas systems are frequently colocalized with the *pAgo* cluster, suggesting their functional synergy^100^. Furthermore, Type I‐F CRISPR‐acquired spacers can be used by Type III‐B systems to enhance the robustness of defense and prevent viral escape^101^.

## EVOLUTIONARY ADAPTATION AND ARMS RACE DYNAMICS

**Figure 4:**
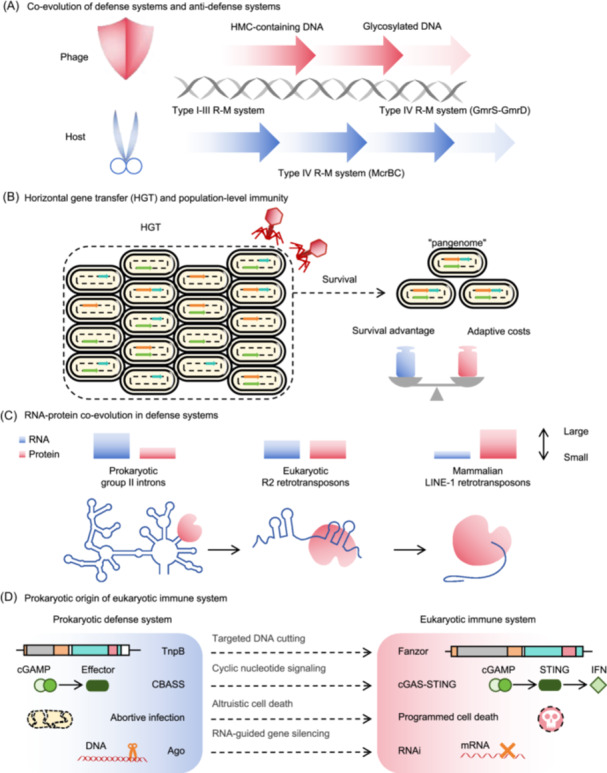
Evolutionary adaptation and arms race dynamics. (A) Co‐evolution of defense and anti‐defense systems. Bacteria and phages engage in an ongoing evolutionary “cold war,” in which bacteria evolve various defense systems to combat phage attacks and phages develop countermeasures to evade these defenses. This arms race drives the co‐evolution of both species. HMC, hydroxymethylcytosine. (B) Horizontal gene transfer (HGT) and population‐level immunity. Bacteria acquire defense systems through HGT, which enhances their survival and increases adaptive costs. Defense genes often cluster in “defense islands,” reflecting their evolutionary adaptation via HGT. This process allows bacterial populations to share defense systems within the “pangenome.” (C) RNA–protein co‐evolution in defense systems. RNA‐guided mechanisms, which are integral to prokaryotic defense systems, highlight the co‐evolution of RNA and proteins. This interaction is evident in prokaryotic group II introns, eukaryotic R2 retrotransposons, and mammalian LINE‐1 retrotransposons, underscoring the transition from RNA to protein dominance in catalytic functions. (D) Prokaryotic origin of ancestral immunity in eukaryotes. The immune mechanisms of eukaryotes are closely linked to those of prokaryotes, with many eukaryotic systems likely inherited from prokaryotic ancestors.

The evolution of prokaryotic defense systems exemplifies the continuous adaptation of life to viral challenges through molecular innovation and diversification. Selective pressure from phage predation has driven the emergence of intricate genetic architectures, such as repetitive sequences, modular proteins, and RNA‐guided machinery, which collectively enhance defense flexibility. These systems evolve under constant arms race dynamics, where each new bacterial strategy prompts viral countermeasures and refines the molecular precision on both sides. Over time, this process shapes prokaryotic genomic diversity and lays the foundation for the complex immune strategies observed in eukaryotes (Figure 4).

### Co‐evolution of defense systems and anti‐defense systems

The relationship between bacteria and phages resembles an evolutionary arms race, with constant attacks, resistance, and adaptation^2^. Bacteria have evolved diverse defense systems^9^, and phages continuously mutate to evade these systems (Figure 4A). This cycle of evolution and counter‐evolution maintains a dynamic balance in long‐term conflicts.

Building on this, prokaryotes have evolved remarkably diverse defense strategies, with most genomes (78%) encoding at least two defense systems^36^. These systems have undergone continuous evolution driven by natural selection, gene mutations, recombination, and HGT. The protective efficacy of a defense system is not absolute but varies with the genetic diversity of both the host and invading elements. For instance, prophages contribute to genomic complexity, whereas certain phage satellites can shift from parasitic to mutualistic states by competing with harmful phages^62^. Elucidating these complex interactions between competition and cooperation offers profound insights into the evolutionary dynamics of host–pathogen relationships.

In response to these host defenses, anti‐defense genes often cluster in viral genomes or MGEs. Phages frequently mutate and acquire anti‐defense proteins to evade the host defense systems. For example, phages have evolved diverse anti‐CRISPR (Acr) strategies with the discovery of Acr factors (Acrs), revealing the complexity of this molecular arms race. Early research focused on protein‐based Acrs^102^, ^103^, whereas a recent study identified rAcrVIA1, an RNA‐based anti‐CRISPR factor (rAcr) that inhibits Cas13 via RNA structural mimicry rather than sequence homology^104^. Mechanistically, Acr proteins can be broadly classified into two categories: (i) target DNA binding inhibitors, which block recognition or crRNA function (e.g., AcrIIA4, AcrIIC3, AcrVA1, AcrVA5, and AcrIF1), and (ii) DNA cleavage inhibitors, which inhibit nuclease activity by binding to catalytic domains (e.g., AcrIIC1 and AcrIE1)^105^. Beyond Acrs, phages also encode sponge‐like proteins (e.g., Tad1, Tad2, Acb2, and Acb4) that sequester second messengers, such as cyclic oligonucleotides or related small signaling molecules, thus neutralizing CBASS, Thoeris, or Pycsar defenses without degrading those signals^95^.

Rearrangement of defense sensors and effectors counteracts the phage evasion. This ongoing adaptation and counter‐adaptation constantly drives co‐evolution. For example, T4 phage DNA with hydroxymethylcytosine (HMC) escapes recognition by cytosine‐based types I–III R–M systems. *Escherichia coli* counters a type IV R–M system (McrBC) that targets HMC‐containing DNA^106^. T4 phages then glycosylate their DNA, inhibiting McrBC activity. This prompts the evolution of the type IV system (GmrS–GmrD), which helps *E. coli* cleave glycosylated DNA^107^. This evolutionary arms race highlights the adaptability and evolutionary dynamics of both phages and bacteria.

As this ongoing co‐evolution continues, researchers have identified new defense systems, such as the phage anti‐restriction‐induced system (PARIS)^59^. The PARIS system, found in ~5.2% of prokaryotic genomes, encounters the T7 anti‐restriction protein Ocr, a phage‐encoded inhibitor of R–M and BREX systems^108^. By sensing Ocr and releasing AriB through the ArA–AriB module, PARIS triggers Abi, preventing phage propagation^59^. However, mutations in Ocr allow phages to escape PARIS and block *Eco*KI^59^. This arms race between prokaryotes and viruses demonstrates an intricate balance of adaptation between defenses and countermeasures, with the emergence of novel viral innovations, such as RNA‐based Acrs and sponge‐like proteins, further highlighting the dynamic co‐evolutionary landscape^109^, ^110^. This interplay shapes evolution and reveals the complexity of microbial survival strategies, emphasizing the delicate equilibrium maintained at the evolutionary scale.

### HGT and population‐level immunity

To survive, bacteria use HGT strategies to acquire various defense systems from MGEs (Figure 4B). MGEs, including phages, conjugative elements, satellites, and mobilizable elements, can move within or between genomes through unknown transfer mechanisms. These systems confer survival advantages but also impose adaptive costs, limiting the number of defense systems that bacteria can maintain, thus forming a co‐evolutionary network with their hosts^111^. Although individual strains may not possess all defense systems, HGT among bacterial populations enables them to indirectly access all defense systems within the “pangenome,” broadening immune protection^10^. Defense genes in bacteria and archaea are often clustered in “defense islands,” reflecting their evolutionary adaptation through the HGT of these systems.

HGT among bacteria, which facilitates the exchange of defense systems, suggests an evolutionary strategy in which collective immunity within bacterial populations takes precedence over individual defense capabilities. This pangenomic sharing highlights the evolutionary trade‐off between acquiring defense systems and managing associated metabolic costs.

### RNA–protein co‐evolution in defense systems

RNA‐guided mechanisms, which are crucial for prokaryotic defense, likely represent ancient evolutionary traits that are foundational to the development of early life forms. These mechanisms are exemplified by two dominant nucleic acid–targeting systems: Ago and Cas proteins. Both systems use RNA‐mediated recognition to neutralize invasive genetic elements, underscoring their indispensable role in combating viral threats throughout evolution.

MGEs such as retrotransposons drive genomic diversification through targeted insertional mutagenesis and sequence expansion. Group II introns, originally identified as mobile retroelements in prokaryotes, are considered the evolutionary ancestors of more complex elements, such as eukaryotic non‐long terminal repeat (LTR) retrotransposons and spliceosome components^112^. These introns have demonstrated the capacity for host defense by disrupting other MGEs, suggesting their potential role in prokaryotic immunity^113^. A comparison of prokaryotic group II introns, eukaryotic R2 retrotransposons, and mammalian LINE‐1 retrotransposons revealed a gradual evolutionary shift in which RNA structural elements were progressively replaced by protein‐coding regions (Figure 4C)^114^. This shift moved from RNA‐based to protein‐based catalytic functions.

This evolutionary transition is further exemplified by CRISPR effectors, which show the co‐evolution of RNA and proteins. Larger Cas proteins correspond to smaller guide RNAs (gRNAs), indicating an inverse relationship^115^. This trend reflects the increasing importance of protein structure and enzymatic function. CRISPR effectors with larger Cas proteins and smaller gRNAs excel in DNA editing, whereas compact CRISPR–Cas systems are suitable for specific applications^116^.

These examples illustrate that RNA and proteins undergo complex interactions and co‐evolution during the evolution of prokaryotic defense systems. In the CRISPR–Cas systems, RNA's catalytic roles of RNA are increasingly dominated by proteins, highlighting their co‐evolution. This shift toward protein dominance boosts the efficacy of immune effectors against these genetic threats.

### Prokaryotic origin of eukaryotic immune system

Evolutionary links exist between the defense systems of prokaryotes and those of eukaryotes. Certain structural domains and proteins originating from prokaryotic defense systems are evolutionarily conserved in eukaryotes and functionally contribute to the diverse stages of innate immunity signaling cascades (Figure 4D)^117^.

Prokaryotic Ago proteins are precursors of the RNA pathway in eukaryotes^118^. TnpB proteins in prokaryotes likely evolved into eukaryotic Fanzor (Fz) proteins^119^. Prokaryotic group II introns are precursors of eukaryotic nuclear splicing introns^120^, ^121^. Similarly, bacterial CBASS systems are considered precursors of the cGAS–STING pathway in animal cells^63^. TIR domains are critical components of immune receptors in bacteria, plants, and animals^122^. cCMP and cUMP, first identified as antiphage second messengers in the bacterial Pycsar system, are co‐opted in mammals to regulate embryonic development (cCMP) and apoptosis (cUMP)^123^, underscoring their evolutionary conservation and functional diversification across domains. SAVED/CARF proteins act as second messenger receptors in both prokaryotes and eukaryotes^124^. This resemblance continues with nonspecific RNase cleavage activation by 2′‐5′‐oligoadenylate synthetase (OAS) in eukaryotes and Type III CRISPR–Cas systems in prokaryotes^63^. The bacterial Abi pathway is analogous to programmed cell death in eukaryotes, and NAD^+^ depletion is a self‐destructive mechanism in both prokaryotes and eukaryotes^125^. The pore‐forming functions of PycC pyrimidine cyclases and gasdermin proteins are similar in prokaryotes and eukaryotes^73^, ^126^. The bacterial Bil system uses ubiquitin‐like protein E1 and E2 conjugating enzymes for antiphage defense^127^. During infection, this system attaches ubiquitin‐like proteins to the phage central tail fiber, disrupting its assembly and reducing its infectivity. This mechanism is analogous to that of the eukaryotic ISG15 system, in which ubiquitin‐like proteins inhibit viral replication. The shared ancestry of prokaryotic and eukaryotic immunity suggests the existence of complex immune systems in higher organisms, which stem from bacterial‐defense strategies. This link underscores the fundamental role of prokaryotic systems in the evolution of the eukaryotic immune system.

## CONCLUDING REMARKS

Prokaryotic antiviral defense systems show remarkable mechanistic and evolutionary diversity, shaped by long‐standing phage arms races. Despite their variety, numerous defenses rely on conserved components, such as nucleases, signaling proteins, or Abi effectors, which are recurrently repurposed across lineages, forming a modular and interconnected immune network rather than a set of isolated systems^65^, ^128^. Comparative studies have uncovered unexpected mechanistic parallels, such as cyclic nucleotide signaling shared by CBASS and Type III CRISPR–Cas, whereas retrons use TA activation strategies^98^, ^129^.

Besides their biological importance, these systems have substantial translational relevance. CRISPR nucleases have revolutionized genome editing^78^, ^130^, whereas retrons, OMEGA nucleases, and cyclic oligonucleotide‐based effectors further enrich the molecular toolkit^93^, ^131^, ^132^. Recently, the concept of “append editing” was introduced, in which the bacterial antiphage toxin DarT2 is harnessed to append ADP‐ribosyl groups to DNA, enabling templated repair in bacteria and base mutagenesis in eukaryotes. By fusing attenuated DarT2 to Cas9 nickase, site‐specific ADP‐ribosylation of thymine residues can be programmatically induced, offering editing outcomes beyond the reach of current base editors^133^. Moreover, the broad repertoire of defense‐associated toxins and signaling enzymes provides promising avenues for developing antimicrobials against MDRs based on the rational exploitation of phage–bacterial interactions^134^.

New discoveries will increasingly rely on AI‐assisted genome mining, terascale clustering, and integrative multi‐omics^135^. Approaches such as Evo genome‐scale modeling and deep learning‐guided inference of defense architectures^136^, ^137^ combined with high‐resolution structural biology^138^ are expected to reveal cryptic or composite systems and illuminate their evolutionary logic. Ultimately, viewing prokaryotic defense through modular and translational perspectives establishes it not only as a historical record of host–virus conflicts but also as a rich source of next‐generation biotechnological tools and anti‐infective strategies^139^, ^140^, ^141^.
